# Theory-based E-health literacy interventions in older adults: a systematic review

**DOI:** 10.1186/s13690-020-00455-6

**Published:** 2020-08-10

**Authors:** Sara Pourrazavi, Kamiar Kouzekanani, Shahrzad Bazargan-Hejazi, Abdolreza Shaghaghi, Mina Hashemiparast, Zahra Fathifar, Hamid Allahverdipour

**Affiliations:** 1 Department of Health Education & Promotion, Tabriz University of Medical Sciences, Tabriz, 14711 Iran; 2 College of Education & Human Development, TAMUCC, 6300 Ocean Dr., Unit 5818, FC 223, Corpus Christi, TX 78412-5818 USA; 3 Department of Psychiatry, Charles R. Drew University of Medicine and Science, Los Angeles, CA USA; 4 Medical Education Research Centre, Tabriz University of Medical Sciences, Tabriz, Iran; 5 Department of Public Health, Maragheh University of Medical Sciences, Maragheh, Iran; 6 Department of Library, Tabriz University of Medical Sciences, Tabriz, Iran; 7 Clinical Psychiatry Research Center, Tabriz University of Medical Sciences, Tabriz, Iran

**Keywords:** E-health literacy interventions, Systematic review, Theoretical study, Elderly, Self-efficacy

## Abstract

**Background:**

e-health literacy can facilitate the uptake of benefits of health for older adults. In this review, we aimed to tabulate the types and outcomes of the theory-based e-health interventions that had been applied to improve the e-health literacy of older adults.

**Methods:**

In this systematic review, theory-based e-health literacy interventions that published up to April 2020 were retrieved from several online electronic databases, including Medline via PubMed, Cochrane Library, ProQuest, and EMBASE. The published papers were included in this study, if the study had been conducted on older adults, a theory-based intervention aimed at promoting e-health literacy, and had been written in English language in the timeframe of 2008–2020.

**Results:**

A total of 1658 records were identified initially, of which, 12 articles met the inclusion criteria. The systematic review identified the using of variety of intrapersonal, interpersonal, and societal level conceptual models in enhancing of e-health literacy in older adults, and the concept of self-efficacy was applied in the most of interventions as the main conceptual theoretical framework.

**Conclusions:**

Despite the paucity of conceptual models, which are specifically designed for e-health literacy interventions, based on our findings, we recommend self-efficacy as a powerful concept that can play an important role in improving e-health literacy in older adults.

## Background

Computer-based electronic health (e-health) literacy has the potential to alleviate barriers for accessing to health care services and facilitating health care delivery [1]. Electronic content within the scope of health sciences could also provide an overwhelming supply of indispensable knowledge for those who are in pursuit of information for better decision-making alternatives [2, 3]. The intrinsic aspect of e-health literacy for older adults, especially those with multiple chronic conditions, physical limitations, and living alone, could be impressive in daily life [4–6]. Older adults, due to physical limitations and constraints in attending face-to-face educational meetings, are likely to benefit from e-health, which in turn may positively effect on their health status and also on their families and communities [5]. However, diversity of e-health literacy level across countries and even inside geographical regions creates major challenges for health care providers and e-content developers [7].

An appropriate and effective exploration of e-health content requires some basic computer literacy and skills, including the know how to operate and maintain a computer, [8]. However, given the variety of e-health information for various users, the ability to search, find, and understand them is essential for becoming self-reliant [9]. Hence, individuals, seniors in particular, who are not computer savvy may face problems to seek or access to the needed health information [10–12].

Consistent with the growth of older people, an augmented demand for the improvement of e-health literacy has been reported, even in developed countries, to facilitate learning and understanding of e-health information [12]. However, it is shown that interventions targeting younger age-groups may not be appropriate for older adults due to cognitive, physiological, environmental, and age-related changes [1]. Additionally, in spite of the proven benefits of e-health literacy interventions among older people [11, 12], they are under-represented in health promotion and disease prevention programs [13].

In addition, during the last three decades, theory-based interventions have had significant roles in shifting research from discovering new facts and explaining events, predicting outcomes, strengthening the efficacy of findings towards successful and efficacious results; thus, behavior modification programs would be effective if and when they are developed based on a suitable theoretical framework [14, 15]. The evidence suggests that theoretically-informed interventions lead to better outcomes [16] because theories present a systematic approach to understanding phenomena by providing explanations for why and under what situations [14].

There are various theories and models that can be used to design effective interventions [14], which are different in complexity and scope of application and have many overlaps with each other. Taking this abundance and diversity of theories and models into account, researchers and practitioners are faced with difficult decisions to choose these theories in practice [17].

Although e-health literacy interventions have benefits for older adults [11, 12], they have not been adequately investigated in aged population. While a previous review provided valuable results in this regard [18], the focuses were not merely on applications of theory-based interventions. In the current systematic review, we have categorized the theory-based interventions about e-health literacy development, and next have explored the theory-based concepts that might be contributed in the effectiveness of the e-health literacy interventions in the elderly.

## Methods

### Study design and search strategy

The present review was conducted according to the Preferred Reporting Items for Systematic Reviews and Meta-Analyses (PRISMA) [19]. The protocol for this systematic review was not registered, but it is available at ResearchGate (https://www.researchgate.net/publication/343135960). Studies were searched using multiple resources to search for available relevant studies, including Medline via PubMed, Cochrane Library, ProQuest, and EMBASE.

We used the study’s research questions to guide the search terms, namely, (a) what theory-based intervention strategies have been used to improve the e-health literacy in the older adult population and (b) what outcome measures have been reported? Therefore, search terms were framed a priori, using Boolean logic. An example of the search strategy for the PubMed database was as follows:

(“Computer Literacy”[Mesh]) OR (“Telemedicine”[Mesh]) OR (internet based health information [Title/Abstract]) OR (internet based health information [Other Term]) OR (internet-based health information [Title/Abstract]) OR (internet-based health information [Other Term]) OR (telehealth literacy [Title/Abstract]) OR (telehealth literacy [Other Term]) OR (mobile health literacy [Title/Abstract]) OR (mobile health literacy [Other Term]) OR (electronic health literacy [Title/Abstract]) OR (electronic health literacy [Other Term]) OR (medical literacy [Title/Abstract]) OR (medical literacy [Other Term]) OR (internet health information [Title/Abstract]) OR (internet health information [Other Term]) OR (computer literacy [Title/Abstract]) OR (computer literacy [Other Term]) OR (online health literacy [Title/Abstract]) OR (online health literacy [Other Term]) OR (online health information literacy [Title/Abstract]) OR (online health information literacy [Other Term]) OR (online health information seeking [Title/Abstract]) OR online health information seeking [Other Term]) OR (web based health information [Title/Abstract]) OR (web based health information [Other Term]) OR web-based health information [Title/Abstract]) OR (web-based health information [Other Term]) AND (theory [Title/Abstract]) OR (theories [Title/Abstract]) OR (model [Title/Abstract]) OR (theory-driven [Title/Abstract]) OR (theoretical model [Title/Abstract]) OR (theoretical study [Title/Abstract]) OR (theory [Other Term]) OR (theories [Other Term]) OR (model [Other Term]) OR (theory-driven [Other Term]) OR (theoretical model [Other Term]) OR (theoretical study [Other Term]) AND (old* adult*[Title/Abstract]) OR (old* people [Title/Abstract]) OR (elderly [Title/Abstract]) OR (aging [Title/Abstract]) OR (aged [Title/Abstract]) OR (ageing [Title/Abstract]) OR (senior [Title/Abstract]) OR (babyboomer [Title/Abstract]) OR (retiree*[Title/Abstract]) OR (pensioner [Title/Abstract]) OR (old* adult*[Other Term]) OR (old* people [Other Term]) OR (elderly [Other Term]) OR (aging [Other Term]) OR (aged [Other Term]) OR (ageing [Other Term]) OR (senior [Other Term]) OR (babyboomer [Other Term]) OR (retiree*[Other Term]) OR pensioner [Other Term]). Moreover, we examined the references of identified publications for relevant studies. This phase of the study screening yielded a total of 1169 articles after removing duplicate papers.

### Study eligibility criteria and selection

The study’s eligibility criteria were formulated, a priori, utilizing the PICO (population, intervention, comparisons, outcomes) framework, and the content validity was examined and approved by members of the research team (SP, ZF, HA):

- *Populations* referred to studies that were included subjects 60 years and older as participants.
- *Interventions* were delimited to those in which the theoretical framework had been explicitly named, referenced, and used. The theory was defined as a set of analytical principles or statements, including defined variables, a domain to which the theory applies, and a set of relationships between the variables and specific predictions [20]. Theory-informed frameworks or models were also considered.
- *Comparisons.* We did not consider any other intervention for comparison.
- *Outcomes* were any reported impact of the theory-based intervention on the improvement of e-health literacy.
- *Time*. All articles published between January 2008 to April 2020 were considered.
- *Setting****.*** There was no limitation based on the type of settings.

The eligible study designs included English language quantitative (randomized controlled trials, non-randomized studies of interventions (NRSI), time series, and before-after studies), or mixed methods (focusing on the quantitative strand) studies. Systematic reviews were not included but used to identify additional eligible studies. Studies that referenced a theory, model, or framework but did not discuss it and/or provided no empirical evidence to support the effectiveness of the intervention were not included in our study.

A final comprehensive search strategy, in accordance with the Peer Review of Electronic Search Strategies statement [21], was developed in consultation with a medical librarian. Two authors independently conducted the search and screened studies for eligibility based on the inclusion criteria, screening of titles and abstracts, and concluded by assessing the full texts of the remaining 19 articles.

### Screening the full-text and synthesis

The selected studies were used to extract the necessary data. Specifically, a data extraction form was developed with input from the research team to collect information on study characteristics, demographic information, aim, theoretical framework, and the use of the theory in identifying determinants, selecting/tailoring interventions, and evaluating the impact of the intervention(s). Additionally, in case of extracting of qualitative details in the review process, the findings were synthesized using a qualitative narrative approach.

**Fig. 1:**
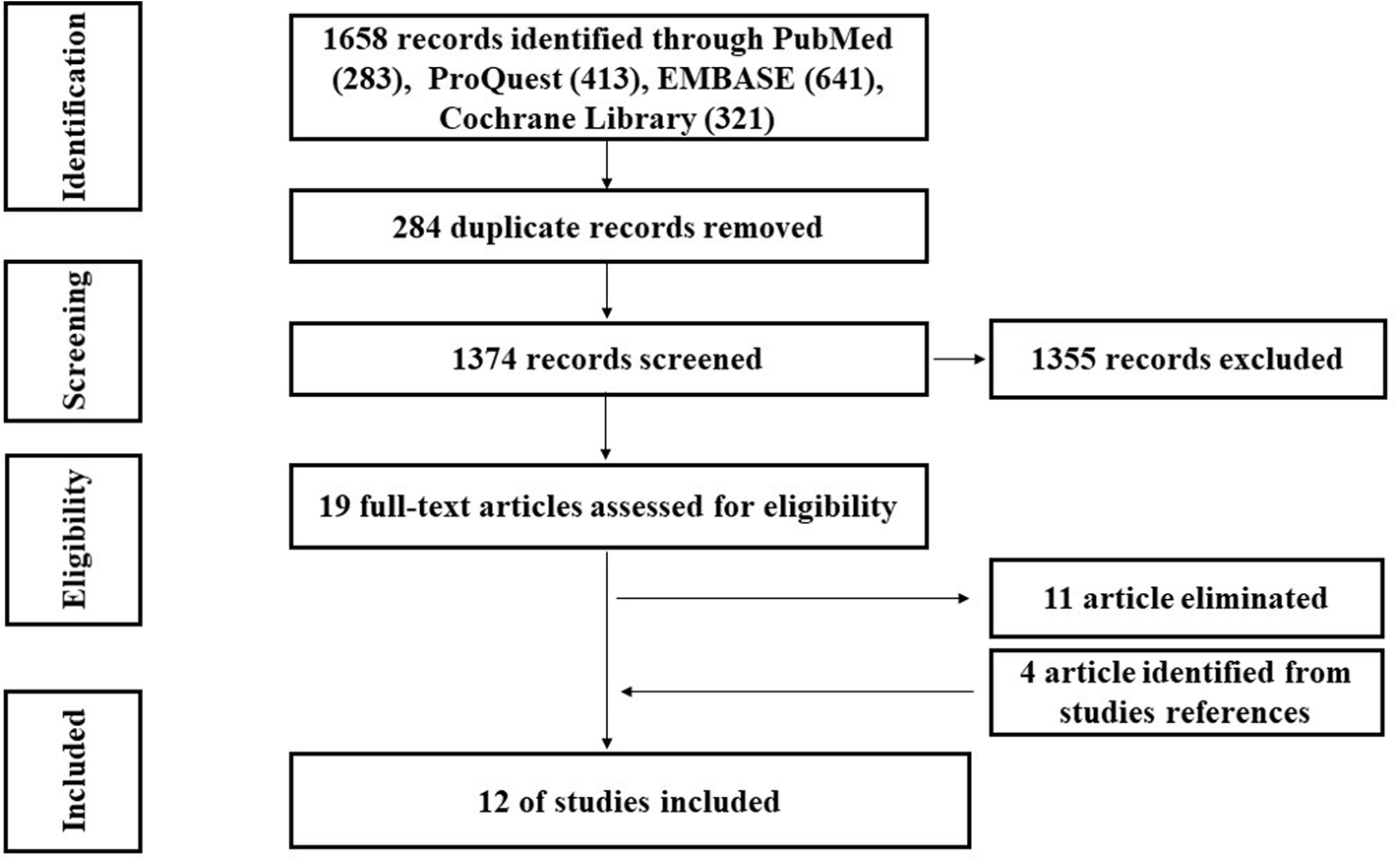
PRISMA Flow Diagram to Detail Study Search Findings

Two members of the research team, SP and ZF, independently pilot-tested the data extraction form, utilizing two of the nineteen articles, compared and discussed the findings, and the feedback was used to refine the form. The final draft of the form was used by SP to extract data from the remaining seventeen articles, which were independently checked by ZF. Next, both of SP and ZF reviewed the full texts of the articles and cross-validated the eligibility based on the aforementioned inclusion criteria. Subsequently, eleven of the nineteen articles was eliminated, resulting in a final sample of eight studies. Articles were eliminated if not reporting empirical data [22, 23], not having a specific theoretical framework [5, 22, 24–28], or not providing information on the intervention content, materials, or results [29, 30]. In addition, Chu’s dissertation and her pilot study [31] were same to Chu et al. (2009) article, therefore we entered just Chu et al. (2009) study [32]. A detailed examination of the references of the eight articles resulted in four additional studies that met the inclusion criteria. The four-round selection process is summarized in Fig. 1. We used the PRISMA flow chart to document and summarize the identification, screening, eligibility, and selection processes. Finally, the twelve articles were independently reviewed by SP and ZF, relevant data were extracted, and if there were any discrepancies, it was discussed until 100% agreement was achieved.

### Quality assessment

The qualitative assessment was conducted independently by two reviewers (SP and ZF) using the CONSORT and TREND checklist. These checklists consist of 25 and 22 criteria, respectively. If a study fulfilled a criterion it received one point. A higher overall score indicated lower methodological bias. The obtained risk of bias score of each study was divided by 25 in CONSORT and 22 in TREND (highest attainable score) and multiplied by 100 to obtain the percentage of fulfilled criteria. The disagreement between the reviewers was resolved through discussion and consensus with a third reviewer (HA). Studies were then grouped into low (> 66.7% fulfilled criteria), moderate (50–66.7% fulfilled criteria), and high risk of bias (< 50% fulfilled criteria) [19, 33].

## Results

### Descriptive findings

Table 1 illustrates the main characteristics of the included studies (*n* = 12). Six studies used a NRSI designs [1, 34, 38, 40, 42, 43]. The sample sizes varied from 11 [38] to 272 participants [41]. The participants’ age ranged from 50 [40] to 101 [39], with the average age ranging between 68 and 75. There were more women than men in nearly all the studies. Data collection in 11 studies took place in the United States [1, 34–39, 41–43], and one study was conducted in Taiwan [40].

**Table 1:** E-health literacy improvement interventions for older adults

Author(s)	Design	Population	Intervention	Intervention Materials	Theoretical framework	Results
**Xi, and Bugg, 2009** [42]	NRSI	131 older adults aged 54–89	8-sessions (twice a week, for 2 hours) training in small groups based on coach education and classroom practice.	Materials developed by the NIA of NIH	Self-efficacy theory	The computer interest and self-efficacy increased significantly from pre- and post-intervention. Computer anxiety decreased after intervention.
**Chu et al., 2009** [32]	RCT	137 older adults aged 65 and older	Training in small groups based on coach education and classroom practice.	Researcher-designed materials	Self-efficacy theory	The computer confidence and self-efficacy increased significantly after the intervention. Computer anxiety decreased after intervention.
**Woodward et al., 2010** [43]	RCT	83 older adults aged 60–89	6-month training program using staff-directed model.	Researcher-designed materials	Self-efficacy theory	The computer self-efficacy, ICT use, and perceived social support increased.
**Xie, 2011a** [34]	RCT	146 older adults aged 56–91	collaborative learning	Materials developed by the NIA of NIH	Self-efficacy theory and Social interdependence theory	Knowledge and skills of website use, and e-health literacy self-efficacy increased.
**Xie, 2011b** [35]	RCT	124 older adults aged 60 and older	collaborative learning	Multimedia tutorial developed by the National Library of Medicine of the NIH	Self-efficacy theory and Social interdependence theory and cognitive theory of multimedia learning	E-health literacy self-efficacy, knowledge and skills of website use increased.
**Xie, 2011c** [36]	NRSI	172 older adults aged 60 and older	collaborative learning	Materials developed by the NIA of NIH	Self-efficacy theory and Social interdependence theory	E-health literacy self-efficacy and skills of participants improved.
**Xie, 2012** [1]	NRSI	218 older adults aged 60–89	8-sessions (twice a week, for 2 hours) training in small groups based on coach education and classroom practice.	Materials developed by the NIA of NIH	Self-efficacy theory	The computer and internet knowledge, interest and self-efficacy increased significantly from pre- and post- intervention. Computer anxiety decreased after intervention. Attitudes improved from pre- and post-intervention.
**Woodward et al., 2013** [37]	NRSI	19 older adults aged 61–85	18-session training using Peer Tutor Model	Researcher-designed materials	Self-efficacy theory	The computer self-efficacy and ICT use increased.
**Cooper-Gaiter, 2015** [38]	NRSI	11 older adults aged 55 and older	5-week (days per week) computer knowledge and skills workshop	Researcher-designed materials	Self-efficacy theory	The computer self-efficacy increased and anxiety decreased after intervention.
**Fink and Beck, 2015** [39]	RCT	65 older adults 50 and older	develop and evaluate a theory-based educational website	Researcher-designed materials	HBM and Knowles theory	Participants assigned higher ratings of usability and learning to the new site, self-efficacy or knowledge didn’t change after intervention.
**Chiu et al., 2016** [40]	NRSI	39 older adults aged 53 to 77	8-sessions training in small groups based on coach education and classroom practice.	Researcher-designed materials	TAM and DO	Computer anxiety decreased and elderly e-health literacy efficacy increased.
**Nahm et al., 2019** [41]	RCT	272 older adults aged 50–92	The 3-week older adult friendly Theory-based Patient portal e-Learning Program	Materials developed in following with NIA guidelines	Self-efficacy theory	Patient portal knowledge, self-efficacy, e-health literacy, health decision making and patient–provider communication improved.

### Risk of bias within studies

On average, the studies fulfilled 65.7% of the assessment criteria (range = 54–81%). Hence, overall the studies had a moderate risk of bias and over half of the studies (*n* = 6 studies) had low risk of bias (range = 68–81%) [1, 34, 35, 38, 40, 41]. The rest of the studies had a moderate risk of bias (range = 54–64%) [36, 37, 39, 42, 43].

### Conceptual frameworks used in e-health literacy interventions

Several general conceptual frameworks were used across the studies to improve e-health literacy in older adults. Specifically, the Health Belief Model (HBM) in health behavior research [39], the self-efficacy theory [1, 32–43], and a combination of Technology Acceptance Model (TAM) and the Diffusion of Innovations model (DOI) [40]. Additionally, two studies used the Social Interdependence Theory [35, 36], one study was guided by Cognitive Theory of Multimedia Learning [36], and another study applied Knowles’ theory as the theoretical framework [39].

### Training tools in e-health literacy interventions

Several intervention tools and/or manuals were used to promote e-health literacy of older adults. Some studies used instructional manual developed by the National Institute on Aging (NIA) of the National Institutes of Health (NIH) [1, 34–36, 41, 43]. Xie (2011) also used a multimedia tutorial developed by the National Library of Medicine of the NIH [36]. Additionally, materials from the NIH Senior Health, namely, Training the Trainers Toolkit, which provide a curriculum for training seniors on how to perform an online search to access reliable health information, had been utilized [18]. The other studies developed their own training materials [37–40, 42].

### Strategies in e-health literacy interventions

Interventions were intrapersonal, interpersonal, or societal, focusing on improving the participants’ e-health literacy via the instructor/teacher-center and/or interactive/collaborative/peer tutor delivery approaches. Five studies used didactic sessions and workshops in classroom settings [32, 38, 40, 42, 43], and three applied collaborative learning and peer tutoring model [35–37]. Fink and Beck (2015) collaborated with a multidisciplinary team of educators, health professionals, and community participants to develop an educational website to improve older adults’ skills in identifying high-quality health information [39]. Nahm et al. (2019) assessed the impact of Theory-based Patient portal e-Learning Program (T-PeP) on patients’ knowledge about electronic portals, health decision-making self-efficacy for being proactive in health communication, patients’ self-efficacy for using electronic portals, and e-health literacy in older adults [41].

Self-efficacy model was the common conceptual model used nearly in all the e-health literacy improvement interventions. For example, the NIA toolkit (http://nihseniorhealth.gov/toolkit/toolkit.html), which was used in the studies conducted by Xie, had a number of features to improve the individual’s self-efficacy in using a computer to access e-health. In Xie’s toolkit, each training session was created based on the information from the previous session and gradually added to the complexity of lessons and goals. Therefore, the knowledge and skills of learners increased gradually during the sessions, which assisted the participants in competency building and computer self-efficacy [1].

Chu et al. (2009) in partnering with seniors for better health program, used instructors to develop older adults’ computer literacy and increase their skills in retrieving and appraising online health information. By breaking the skills to simpler steps and gradually adding more complex steps, instructors not only enhanced the participants’ self-efficacy, but also enabled them to perform a more complex Internet search for accessing e-health information [32].

Fink and Beck used the constructs of HBM to develop e-Health literacy by improving the online searches abilities. For example, they increased the users’ perceived threat by demonstrating the pitfalls of relying on public search engines. To increase the participants’ self-efficacy, they provided user-friendly guidelines for evaluating websites quality, provided a list of high-quality websites, and illustrated the benefits of using high-quality websites [39].

Cooper-Gaiter (2015) organized a facilitator-led computer workshop and training modules at a local community center. After the workshop, she obtained favorable results in increasing participants’ self-efficacy in using e-health information [38].

In addition, Nahm et al. (2019) emphasized on four sources of efficacy beliefs, namely, (1) successful performance of the behavior, (2) social persuasion, (3) modeling others’ successful performances, and (4) relief of emotional stress [14], which were developed by using various components of T-PeP, such as discussion forums and web modules (text, video, and pictures). For example, participants were encouraged to set goals according to the content they learned through modules and shared their success stories and/or challenges on the discussion board. Moreover, they were informed of potential difficulties with technology in the modules and were provided help desk support [41].

Based on another approach, Bo Xie (2011) used the social interdependence theory as a conceptual framework to improve the e-health literacy of the older adults by developing collaborative learning tactics [34–36]. Collaborative learning is known as one of the most common approaches to active learning [44].

Finally, Chiu et al. (2016) used TAM and DOI to enhance the perceived usefulness of internet technology in accessing e-health. To increase older adults’ acceptability of e-health and reduce their perceived barriers in utilizing complicated computer application, they selected and downloaded several simple e-health computer applications. In addition, they provided a step-by-step training illustrations which indicating the applying of self-efficacy development in this program [40].

## Discussion

Several studies showed an improvement in the older adults’ level of interest, confidence, and self-efficacy in using e-health information [1, 32, 37, 38, 40–43]. Other studies reported an increase in the participants’ web-based e-health knowledge [34–36, 41], access to e-health information and communication technologies [42], e-health literacy [35, 36, 40, 41], and decline in the elderly’s anxiety in using computer or Internet [32, 34, 38, 40, 43].

We also reviewed the conceptual models used in designing e-health literacy interventions in older adults. Among the existing health behavior models, self-efficacy was the most frequently cited conceptual model. However, none of these studies used the conceptual frameworks that were specifically designed for e-health literacy intervention, such as the Lily model or the framework proposed by Chan and Kaufman [8, 45]. This is consistent with the findings of Watkins and Xie [18]. These two models can be useful in explaining e-health literacy and designing tools in this context. However, as Watkins and Xie (2014) stated, the models presented by Lily and Chan and Kaufman (2011) are newly developed and they lack sufficient empirical validation [18].

Self-efficacy refers to a personal belief in his or her capacity to perform behavior/behaviors that is/are necessary to attain specific performance [46]. According to social cognitive theory, self-efficacy beliefs are the central mechanism of human agency. Bandura (1977) stated all behaviors are rooted in the beliefs that one has the power to make the desired changes and the other factors for behavior change may serve as a guide. From his point of view, self-efficacy is a superior predictor of amount of behavioral improvement [46]. The studies that used ‘self-efficacy’ model concluded that once the participant’s self-efficacy in using e-health is enhanced, they are more likely to report positive outcomes in managing their health-related concerns [26, 30, 47–50].

Watson believed that one of the factors that might affect to use less the online health information by older adults, is the lack of internet self-efficacy [51]. Internet self-efficacy also is positively related to performance and intention of older adults for learning of Information and Communications Technologies [52]. Moreover, research has shown that Internet self-efficacy could foster better information searching strategies and learning outcomes in Internet-based environments [53].

In addition, it is postulated that personal self-efficacy beliefs can be improved via one’s sources of information regarding the establishment, reinforcing or weakening of such beliefs, potentially influencing individual’s behavior [46]. Bandura suggested four methods for promoting self-efficacy, namely, (1) mastery experience, (2) social modeling, (3) improving physical and emotional states, and (4) verbal persuasion [14]. Therefore, various strategies can be used to increase computer and internet self-efficacy, which may ultimately result in improving e-health literacy. Increasing health communicating skills through the internet, using videos or images that show different stages of computer and internet use, providing social support, and motivating older adults can be instrumental in positively affecting self-efficacy and eventually improving elderly’s e-health literacy.

The main source of self-efficacy is mastery experience [54]. Therefore, seniors who have little experience in using computers and the Internet, or are unable to make optimal use of the Internet due to lack of education, technical skills, and access to technology, stay away from its usefulness of ICT. However, the older adults are shown to be interested in learning of practices if mastering the task at hand. According to Chu et al. (2009), a combination of patience, perseverance, and peer-to-peer or instructor encouragement has the potential to successfully reduce older adults’ stress and anxiety in learning and raising their self-efficacy [32]. For this reason, training designed by strategies that increase the Internet and computer self-efficacy is effective in the elderly.

### Limitations

This systematic review’s delimitations and limitations must be acknowledged. The sample was delimited to articles published in English, so it may not reflect findings of studies that were published in other languages. The keyword search was restricted to the title, keywords, and abstract for each article. Due to the high variability in the intervention tools, strategies, and outcome, it was not possible to perform a joint statistical analysis of the data. Consequently, a narrative analysis was conducted, which limited the external validity of the findings.

## Conclusion

The aforementioned findings suggest that the application of the conceptual frameworks in the context of e-health literacy interventions has the potential to enhance the outcome measures. Meanwhile, they suggest a need to empirically test the efficacy of the conceptual models that are designed to specifically improve e-health literacy. Based on our findings, we recommend self-efficacy as a powerful concept that can play an important role in improving health literacy in older adults by increasing their confidence in themselves and their abilities to use the Internet and web-based learning despite their age-related limitations.

## Data Availability

Please contact the corresponding author for data requests.

## Abbreviations

PRISMA: Preferred Reporting Items for Systematic Reviews and Meta-Analyses
NRSI: Non-randomized studies of interventions
HBM: Health Belief Model
TAM: Technology Acceptance Model
DOI: Diffusion of Innovations model
NIA: National Institute on Aging
NIH: National Institutes of Health
T-PeP: Theory-based Patient portal e-Learning Program
ICT: Information and Communications Technology
